# Deterministic resonance fluorescence improvement of single quantum dots by optimized surface passivation

**DOI:** 10.1038/s41377-025-01838-6

**Published:** 2025-04-22

**Authors:** Junyi Zhao, Runze Liu, Gengyan Zou, Zhenxuan Ge, Qihang Zhang, Yukun Qiao, Xing Ding, Guoqiu Jiang, Yiyang Lou, Yongpeng Guo, Tunghsun Chung, Yuming He, Chaoyang Lu, Yongheng Huo, Jianwei Pan

**Affiliations:** 1 Hefei National Research Center for Physical Sciences at the Microscale and School of Physical Sciences, University of Science and Technology of China, Hefei, 230026 China; 2 Shanghai Research Center for Quantum Science and CAS Center for Excellence in Quantum Information and Quantum Physics, University of Science and Technology of China, Shanghai, 201315 China; 3 Hefei National Laboratory, University of Science and Technology of China, Hefei, 230088 China; 4 Department of Physics, The Chinese University of Hong Kong, Shatin, New Territories, Hong Kong SAR, China

**Keywords:** Quantum dots, Optical spectroscopy

## Abstract

The degradation caused by surface states restricts the performance of near-surface semiconductor quantum dots (QDs). Here, we demonstrate optimized passivation techniques to improve the resonance fluorescence (RF) with dot-to-dot comparisons. These optimized techniques, for the first time, reduce the linewidth and noise level of existing pulsed-RF signals, as well as revive pulsed-RF signals which originally are vanishing. The improvements are confirmed to originate from reduced surface state density and electric field after passivation, through optical and surface science characterizations. Our study promotes applications of the passivation techniques in thin-film quantum devices, paving the way for the further development of optimal QD-based quantum light sources.

## Abstract (web summary)

Optimized surface passivation deterministically enhances near-surface quantum dots’ resonance fluorescence from disappearance to improved emission.

## Graphical abstract

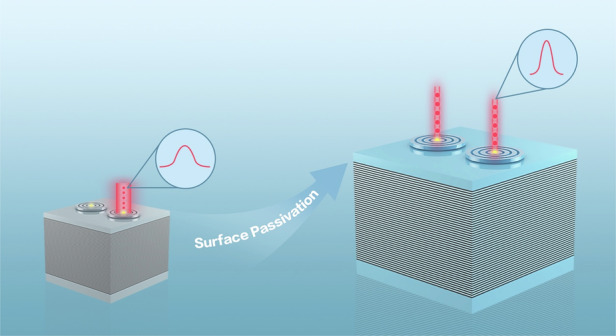

## Introduction

Surface states are inherent limiting factors that degrade the performance of solid-state semiconductor devices, including both classical^1^ and quantum^2^ systems. To address this challenge, surface passivation techniques have been developed and become increasingly significant in modern semiconductor technology^3,4^. This is particularly crucial for quantum devices, as source regions are often closer to the surface, making them more vulnerable to surface effects^5^. Consequently, it is vital to further enhance passivation techniques and to rigorously evaluate them from a comprehensive perspective.

On-demand single and entangled photons are fundamental resources in quantum information processing, making high-quality quantum light sources crucial^6^. Semiconductor QDs have emerged as one of the most promising candidates for quantum light sources^7–11^. With the rapid development of QD-based quantum light sources and scalable quantum photonic circuits^12–14^, more compact device structures are desired to enable broadband emission out-coupling, heterogeneous integration^15^, and loss reduction^16^. For example, QDs often locate at ~60 nm away from the surface when coupled with photonic crystal^17^ and circular Bragg grating^9^ (CBG) cavities. However, surface states degrade the performance of semiconductor devices in such thin regions. For example, surface states introduce obvious linewidth broadening for near-surface QDs^18^.

To overcome this problem, many passivation techniques have been employed. Most of them are demonstrated by comparisons of non-resonant photoluminescence (PL) of QD ensembles^19–22^. However, pulsed-resonance excitation on individual QD is the practical on-demand excitation technique that has been widely applied in quantum information technologies^23–25^. Thus, investigating and improving pulsed-RF signals of near-surface QDs through passivation shows direct value for further development of QD-based quantum light sources.

Here, we faithfully demonstrate the passivation improvements on pulsed-RF signals of individual QDs, utilizing optimized sulfur-based passivation techniques combined with dot-to-dot comparisons. We qualitatively confirm the passivation improvements on non-resonant PL, consistent with previous works^19–22^. More importantly, we quantitatively characterize the resonant PL performance. Our results show that the optimized passivation techniques can reduce the linewidth and noise level of QDs with existing pulsed-RF signals, as well as revive pulsed-RF signals of QDs which originally showed no RF. These improvements can be widely observed. Furthermore, we reveal the mechanism of passivation improvements, which stem from reduced surface state density and electric field, by optical measurements, X-ray Photoelectron Spectroscopy (XPS), and Raman spectroscopy. Our results prove the effectiveness of sulfur-based passivation techniques in the RF region of QDs.

## Results

Our sample is grown by the molecular beam epitaxy (MBE) system. The structure consists of 30 pairs of λ/4-thick AlAs/GaAs distributed Bragg reflector (DBR) and a λ-thick GaAs layer at the top. A layer of self-assembled InAs/GaAs QDs is inserted in the middle of the λ-thick GaAs layer. To investigate the influence of surface states, we etch the surface to let the dot-to-surface distance be less than 40 nm^19^. However, the collection efficiency drops significantly due to the broken symmetry of the source region, making it difficult to collect RF signals. To improve the collection efficiency, we design a hybrid structure combining the advantages of DBR for vertical reflection and CBG for lateral diffraction. The DBR-CBG structure enhances device efficiency and makes it more appropriate for quantitative comparison than solid immersion lenses^26,27^ (SILs) or other optical microcavities^28^.

**Fig. 1:**
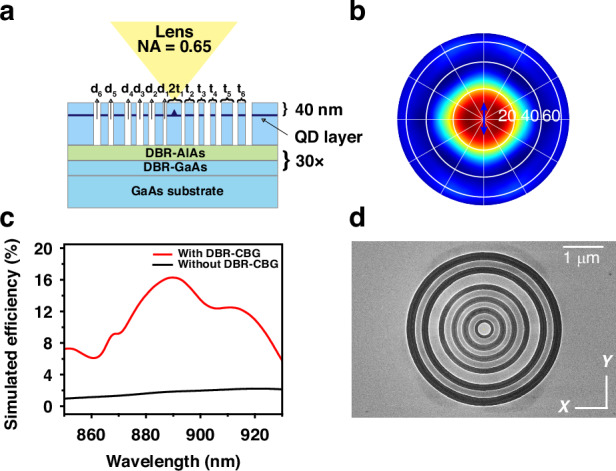
The schematics and illustrations of the DBR (distributed Bragg reflector)-CBG structure. **a** Schematic diagram of the DBR-CBG structure. **b** Numerical simulation of the far-field distribution of the electrical intensity of emission. The blue arrow denotes the dipole orientation. **c** Simulations with and without the DBR-CBG, demonstrating an 8.81-fold improvement in collection efficiency. **d** Top view of a scanning electron microscope (SEM) image of a single DBR-CBG structure

As illustrated in Fig. 1a, Our DBR-CBG structure consists of 6 lateral circular gratings periods with 12 width parameters and 30 vertical pairs of λ/4-thick AlAs/GaAs DBR. The choice of six periods is the balance between improvement in collection efficiency and decrement in fabrication uncertainties. The 12 width parameters of CBG are optimized by the bound optimization by quadratic approximation (BOBYQA) gradient algorithm to maximize the collection efficiency. The numerical results (Fig. 1b) show that the single-photon emission from the DBR-CBG structure is symmetric and directional. Although the simulated Purcell factor is 2.29 and the quality factor (Q) is 53, the enhancement in collection efficiency is still considerable. As can be seen from Fig. 1c, the collection efficiency at 890 nm increases about 8.81-fold to 16.28% with the DBR-CBG structure, enough for acquiring RF signals. The SEM image of the structure is shown in Fig. 1d. Note that every period contains 14 DBR-CBG structures with a location mark, making it locatable to do dot-to-dot comparisons. Details about the DBR-CBG structure are demonstrated in Supporting Information I. The sample growth and fabrication details are shown in Supporting Information II.

An effective passivation should eliminate the surface dangling bonds and protect the passivated surface from re-degradation simultaneously. Hence, we optimize the conventional passivation techniques with a customized passivation system and a two-step process. Our customized passivation system consists of a glove box connected to an atomic layer deposition (ALD) system. The glove box provides an inert atmosphere (H_2_O and O_2_ < 1 ppm), which can prevent the reoxidation of the sulfur layer before ALD deposition. The two-step passivation starts by filtering the (NH_4_)_2_S aqueous solution with 0.02-um syringe filters in the glove box to remove polysulfide particles. After that, we immerse the sample in 20% (NH_4_)_2_S solution for 10 min and subsequently transfer it to the load-lock chamber of the ALD to deposit 10 nm Al_2_O_3_ at 150 °C. Because the transfer is performed under an inert atmosphere, our method eliminates surface dangling bonds and protects the passivated surface from degradation simultaneously. The whole system and process guarantee stable and uniform passivation layers, making our experiment results robust and reproducible. The schematic of our customized passivation system and details about our optimization process are illustrated in Supporting Information III.

**Fig. 2:**
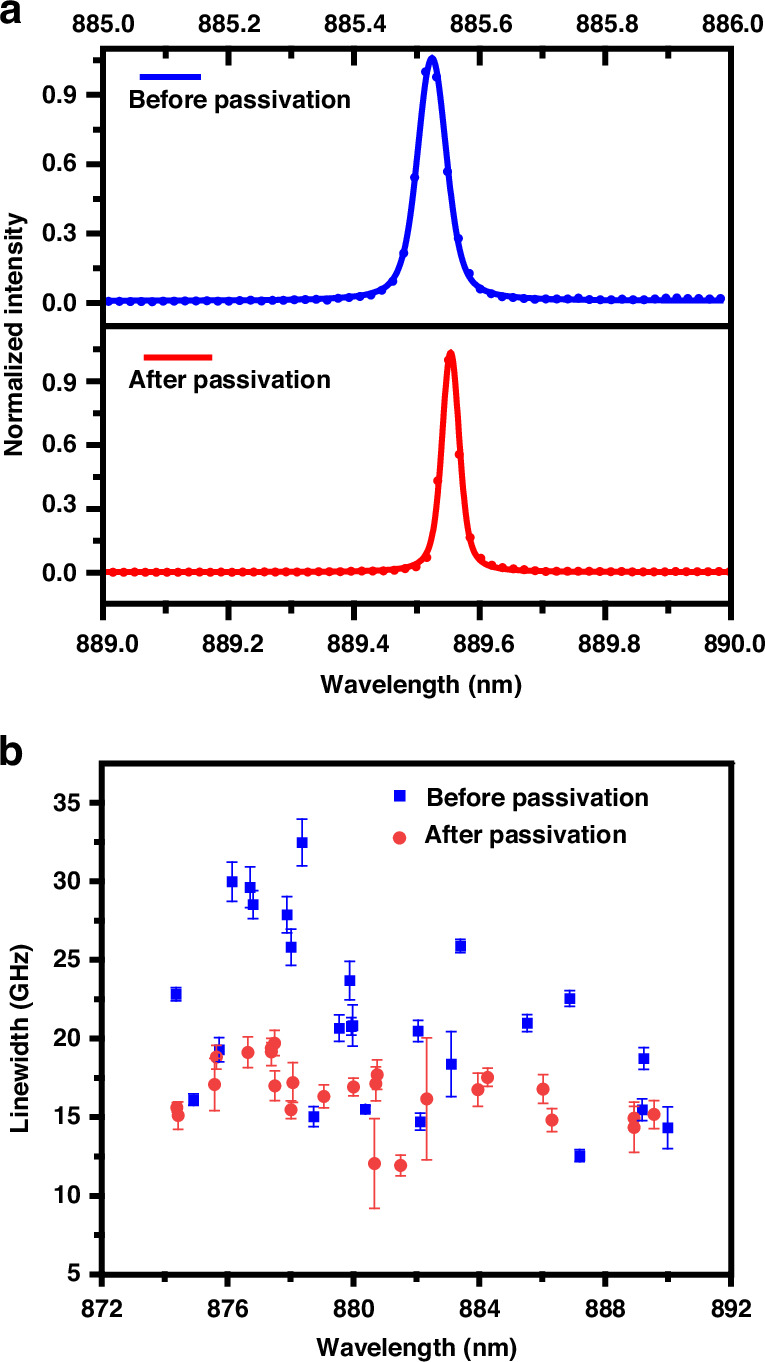
Improvements in the non-resonant optical properties of QDs through surface passivation. **a** PL spectra of the QD representing the typical emission before and after passivation. **b** Comparison of linewidths from 25 randomly selected QDs before and after passivation. All original PL spectra are shown in Supporting Information IV

Based on our DBR-CBG structure and optimized passivation technique, we first qualitatively confirm the passivation improvements with non-resonant PL. To do so, we randomly select 25 QDs from the same sample before and after passivation and compare the non-resonant PL linewidth distribution. The results are shown in Fig. 2a, b. Figure 2a illustrates the typical PL spectra before and after passivation, demonstrating a 39.88% reduction in linewidth after passivation. Figure 2b further illustrates the linewidth distribution of all the randomly selected QDs, the average linewidth decreases from 21.32 ± 5.48 GHz to 16.49 ± 2.03 GHz after passivation. These results, proving our passivation improvements, are consistent with previous works^19–22^.

**Fig. 3:**
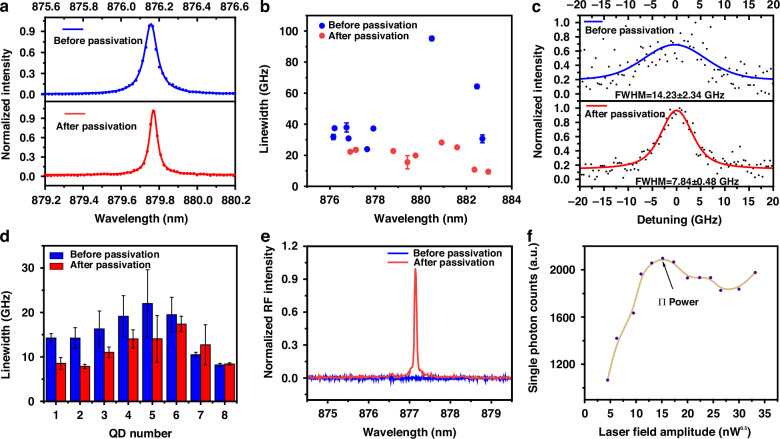
Improvements in the resonant optical properties of QDs through surface passivation. **a** RF spectra of the QD representing the typical emission before and after passivation. **b** Comparison of linewidths from randomly selected QDs before and after passivation under resonant excitation. **c** The fine structure (linewidth) of the pulsed-RF from a typical QD (named after QD2) before and after passivation. **d** Comparison of linewidths from eight individual QDs before and after passivation with dot-to-dot comparisons. All the linewidths are measured by the Scanning Fabry–Pérot (SFP) method. **e** Pulsed-RF spectrum of a typical QD (named after QD-A) before and after passivation. The spectrum revives from mere background to obvious signal after passivation. **f** By varying the amplitude of the pumping laser field, a Rabi oscillation up to 2π is observed

We would highlight again that pulsed-resonant excitation is the practical on-demand excitation technique and pulsed-RF signals are of great significance^23–25^. Thus, we first qualitatively confirm the passivation improvements with RF using a spectrometer. Similar to Fig. 2, we randomly select nine QDs from the same sample before and after passivation and compare the RF linewidth statistical distribution. The results are shown in Fig. 3a, b. Figure 3a illustrates the typical RF spectra before and after passivation, demonstrating a 46.77% reduction in RF linewidth through passivation. Figure 3b further illustrates the linewidth distribution, the average RF linewidth decreases from 43.23 ± 22.53 to 19.68 ± 6.48 GHz after passivation. Note that the average center wavelengths lie in 878.57 ± 2.62 and 880.76 ± 3.36 nm before and after passivation, respectively, showing no apparent overall shifts.

We then step forward to examine the passivation improvements on pulsed-RF quantitatively using dot-to-dot comparisons. Firstly, we confirm passivation improvements on the linewidth of RF. Here, we perform dot-to-dot comparisons on eight QDs. We record the linewidth of pulsed-RF spectra of eight QDs before and after passivation, respectively, utilizing a Fabry–Pérot cavity for higher resolution. The result is that our passivation techniques can reduce the linewidth of pulsed-RF signals. As shown in Fig. 3c, the linewidth of QD2 before passivation is 14.23 ± 2.34 GHz with a low signal-to-background ratio (SBR^29^) and reduces to 7.84 ± 0.48 GHz with increased SBR (detailed discussion in Supporting Information VI) after passivation, demonstrating substantial improvement. Meanwhile, the variance of the photon number fluctuations decreases from 0.2749 to 0.1587 after passivation, indicating a 42.27% reduction in the noise level of QD2 (Supporting Information V). We emphasize that the improvement can be widely observed, as illustrated in Fig. 3d. Most of the QDs demonstrate reduced linewidths after passivation, which proves the passivation validity. We also notice that the RF linewidth of QD7 and QD8 become larger after passivation. We attribute it to newly generated defects around the QD because of (NH_4_)_2_S etching, which is discussed in detail in Supporting Information VI.

Secondly, we want to confirm our passivation improvements on the revival of RF. Before passivation, a high density of surface states arising from the etched surface can bend the energy band, making the electrons tunnel out before radiative recombination happens. That’s the reason why only very small amounts of QDs can emit RF signals, while most of them don’t have RF signals even with non-resonant ancillary lasers before passivation. Thus, surface passivation can revive previously disappeared RF signals related to surface states. To confirm this, we randomly select five QDs that cannot generate RF signals before passivation and recheck after passivation. Our results show that RF can be revived through our passivation techniques on two QDs (one named after QD-A). As shown in Fig. 3e, QD-A exhibits merely background even with additional non-resonant ancillary laser excitation (two-color excitation). After passivation, a bright and sharp line proves undoubtedly the pulsed-RF signals. To confirm the coherent manipulation of the two-level system within the QD, we measure the Rabi oscillation of QD-A and observe the Rabi oscillation up to 2*π* under pulsed-resonant excitation (Fig. 3f).

## Discussion

Based on our experimental improvements in the optical properties of QDs’ luminescence, we now move forward to investigate the mechanism of these improvements in linewidth. As reported, surface states are inherent limiting factors that degrade the optical properties of near-surface QDs. These surface states mainly come from native oxides^30^ as well as nano-fabrication-induced defects^22^ and can be partially eliminated after passivation. Previous works^19–22^ have demonstrated passivation improvements in non-resonant optical properties, but explanations of passivation improvements are not established comprehensively from the experimental perspective. Here, we propose our explanation and verify it from both the theoretical and experimental perspectives. We first suppose our passivation mechanism is as follows: The surface states act as defect centers to trap and de-trap charges. These fluctuating processes induce a fluctuating band-bending of band edges and surface electric field^19^. The fluctuating electric field results in linewidth broadening over longer timescales due to the DC-stark effect^31^. After passivation, the reduced surface states result in a decreased band-bending, electric field, and noise level, which improves the optical properties of near-surface QDs.

**Fig. 4:**
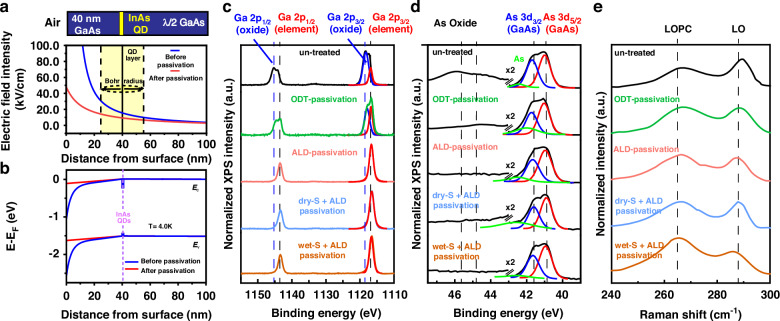
Theoretical and experimental illustrations on the mechanism of passivation improvements. **a** Schematic of near-surface sample structure and corresponding simulated surface-induced electric field versus distance from the sample surface. The range of the Bohr radius^41^ is marked in yellow, and the electric field intensity decreases significantly after passivation. **b** Simulation of the conduction and valence bands in the structure at T = 4.0 K. The surface band-bending decreases significantly after passivation. **c** Ga 2*p*, **d** As 3*d* XPS spectra, and **e** Raman spectra of GaAs (001) surface treating under different passivation techniques. The “untreated” is the reference group. The ODT (1-Octadecanethiol) passivation is conducted through immersion in a 0.05 mol L^−1^ ODT solution. The ALD passivation is performed by 10 nm Al_2_O_3_ coating using ALD. “wet-S” and “dry-S” passivation are achieved through immersion in (NH_4_)_2_S solution and exposure to vapor phase H_2_S, respectively. The “wet-S + ALD” group uses the same passivation techniques in this article, demonstrating the best improvements compared to other techniques. Details concerning four passivation techniques are illustrated in Supporting Information IX

Given this proposed explanation, we examine it from both theoretical and experimental perspectives. Firstly, we confirm it theoretically. According to our explanation, the surface band-bending and electric field should be reduced after passivation. So, we model and simulate surface-induced band edges and electric field of GaAs surface before and after passivation (Supporting Information VII), and the results are illustrated in Fig. 4a, b. As can be seen from the figures, the surface band-bending, as well as the electric field decreases obviously after passivation. We also notice that surface states will not influence the QDs once the dot-to-surface distance is larger than ~100 nm, according to Fig. 4a, b. To confirm this, we perform a dot-to-dot comparison on QD at a depth equal to 127 nm. The results are illustrated in Fig. S11. The RF linewidth equals 1.16 ± 0.02 GHz before and 1.15 ± 0.01 GHz after passivation, demonstrating no apparent change, aligning with our simulations. Meanwhile, the wavelength shifts of QD (Supporting Information VIII) and the improvement in the ratio of 2 Raman peaks (Fig. 4e and Supporting Information IX) are both accurately predicted by our simulated surface electric field before and after passivation, all those results together, make the model convincing.

Secondly, we verify our explanation experimentally. To achieve this, we perform XPS and Raman spectroscopy on GaAs (001) surface with (“wet-S + ALD”) and without (“untreated”) passivation. XPS analysis can study changes in the content of surface oxides, one of the main sources of surface states. Raman measurements can determine changes in surface depletion layer thickness caused by surface states, and thus analyze changes in surface state density^32,33^. To evaluate the validity of our passivation techniques, we also treat samples with other passivation techniques (ODT, ALD, and dry-S + ALD) for comparison^34–36^. XPS shown in this work is taken with monochromatic Al Kα radiation (1486.6 eV) with the anode operating at a power of 150 W, and the binding energy is corrected by the C 1*s* peak at 284.8 eV (Axis Ultra DLD). PL-Raman spectra are recorded at room temperature using 325 nm laser excitation, and the acquisition time is set at 3 s (HORIBA laRAM HR Evolution).

The surface-sensitive Ga 2*p* and As 3*d* XPS spectra of the GaAs surface under different passivation techniques are shown in Fig. 4c, d, respectively. Peaks at 1143.2, 1116.9, 41.6, and 40.9 eV correspond to the GaAs bulk signals, and peaks at 1145.2, 1118.7, 45.6, and 44.8 eV correspond to the native oxide. Surface oxides are observed clearly before passivation. ODT passivation partially eliminates Ga and As oxides, only the process containing ALD passivation completely removes Ga and As oxides, demonstrating good passivation effects. The Raman spectra of the GaAs surface under different passivation techniques are shown in Fig. 4e. The peak near 265.0 cm^−1^ corresponds to the longitudinal-optic-phonon-plasmon coupled (LOPC) mode from GaAs bulk, where the LO phonons couple with the free-electron plasmons. The peak near 288.0 cm^−1^ corresponds to the longitudinal-optic (LO) mode from the surface depletion layer, where there are no free electrons for screening^37^. Therefore, effective passivation results in a larger value of I_LOPC_/I_LO_, representing a better passivation effect (principle and detailed analysis in Supporting Information IX). For the untreated GaAs surface, the ratio of I_LOPC_/I_LO_ is 0.798, standing for high surface state densities. After ODT, ALD_,_ and dry-S + ALD passivation, the intensity of the LOPC peak increases slightly, indicating partially reduced surface states. But under wet-S + ALD passivation (our techniques), the ratio of I_LOPC_/I_LO_ increases up to 2.675, showing the largest increment. The 3.35-fold increment in I_LOPC_/I_LO_ is consistent with the simulation prediction. Consequently, the proposed passivation mechanism about reduced surface states is strongly proved with experimentally observed reduced surface oxides and thinner surface depletion layer. Furthermore, the wet-S + ALD passivation demonstrates till now the best performance compared to other commonly used techniques under XPS and Raman analysis. To highlight the progress, we have summarized the results of other reported experiments^19–22^, which have promoted the study of passivation, as shown in Table 1.

**Table 1:** Comparison of our passivation results with previous works

	Dot-to-dot comparison	Non-resonant PL improvement	RF improvement	Noise reduction	XPS spectra	Raman spectra
This work						
Ref. ^19^ and Ref. ^20^						
Ref. ^21^						
Ref. ^22^						

In summary, we quantitatively study passivation improvement on near-surface QDs in both the resonant and non-resonant regions. With our optimized in situ two-step passivation techniques, we, for the first time, reduce the linewidth and noise level of existing pulsed-RF signals and revive previously disappeared pulsed-RF signals. We also conclude that the improvements from passivation stem from a reduction in surface state density and corresponding surface electric field in the vicinity of the QD region. This conclusion is verified through both the optical and surface science measurement results. Our results provide a comprehensive understanding of the passivation improvements and promote further applications of passivation techniques in thin-film quantum devices, paving the way for the future realization of optimal QD-based quantum light sources and future integrated quantum photonic devices.

## Materials and methods

### Sample design and fabrication

We simulate the light field emitted by a QD based on the finite element method (FEM) in the frequency domain. The QD is modeled as an oscillating dipole to solve the collection efficiency of an objective with a 0.65 numerical aperture. The lateral CBG comprises 6 circular gratings with widths of {2t_1_, t_2_, …, t_6_} separated by six gaps with widths of {d_1_, d_2_, …, d_6_}. All 12 parameters are free parameters, and initial parameters are set based on experience. The 12 width parameters of CBG are optimized by the BOBYQA gradient algorithm to maximize the collection efficiency^38,39^.

The fabrication process consists of three main steps: sample growth, wet etching, and pattern definition. First, we grow the epitaxial structure on a 3-inch GaAs (001) substrate, beginning with 30 pairs of 1 nm AlAs/3 nm GaAs super-lattice to obtain a smoother surface. Next, we grow 30 pairs of λ/4-thick AlAs/GaAs DBR to enhance longitudinal reflection. At last, we grow the source region composed of a λ-thick GaAs film with a layer of self-assembled InAs/GaAs QDs inserted in the middle.

After sample growth, we etch the surface GaAs to let the dot-to-surface distance be less than 40 nm, utilizing a solution with a mixture of H_2_O:H_2_SO_4_:H_2_O_2_ (volume ratio=50:1:1, etch rate ~1.1 nm s^−1^). Subsequently, we define CBG patterns with electron-beam lithography (EBL) and inductively coupled plasma (ICP) etching. Our sample consists of periodically arranged arrays of patterns, and each pattern contains 2 × 7 CBG structures with a location mark. With the marks and the micro photoluminescence setup, we can perform dot-to-dot comparisons.

### Optical measurements and data analysis

We perform optical measurements based on a confocal microscope system^40^. The QD sample sits inside a stable cryostat with an average temperature of 4.0 K. A confocal microscopy is employed to excite the QD, obtain real-time images, and collect emitted single photons simultaneously. The 780 nm continuous wavelength laser is applied for non-resonant excitation. The wavelength-tunable pulsed laser is applied for resonant excitation. To determine the fluorescence linewidth precisely, we send pulsed-RF signals into a home-built Fabry–Pérot cavity. All original data were presented in Supporting Information IV–VI.

## Supplementary information

Supporting Information for Deterministic resonance fluorescence improvement of single quantum dots by optimized surface passivation

## Data Availability

The detailed sample design, growth, fabrication, and passivation process, original photoluminescence spectra as well as linewidth data before and after passivation, analysis of passivation effects on the wavelength of photoluminescence, and analysis of Raman spectra results are included in Supporting Information. This material is available free of charge via the Internet at https://www.nature.com/lsa/articles. All data were true and reliable. Upon a reasonable request, it may be obtained from the corresponding author.
