# Supplementary material for: Deterministic resonance fluorescence improvement of single quantum dots by optimized surface passivation
Source: Light Sci Appl. 2025 Apr 22;14:170. doi: 10.1038/s41377-025-01838-6 (PMC12015415; doi:10.1038/s41377-025-01838-6)
Supplement: Supplementary file 1 — Supporting Information for Deterministic resonance fluorescence improvement of single quantum dots by optimized surface passivation [file 41377_2025_1838_MOESM1_ESM.pdf]

Supporting Information for

Deterministic resonance fluorescence improvement of single quantum dots by optimized surface passivation

Junyi Zhao[1,2,3#], Runze Liu[1,4#], Gengyan Zou[1,2#], Zhenxuan Ge[1,2#], Qihang Zhang[1,2], Yukun Qiao[1,2], Xing Ding[3], Guoqiu Jiang[1,2], Yiyang Lou[1,2], Yongpeng Guo[1,2], Tunghsun Chung[3], Yuming He[1,2,3*], Chaoyang Lu[1,2,3], Yongheng Huo[1,2,3*] and Jianwei Pan[1,2,3]

1. Hefei National Research Center for Physical Sciences at the Microscale and School of Physical Sciences, University of Science and Technology of China, Hefei 230026, China;

2. Shanghai Research Center for Quantum Science and CAS Center for Excellence in Quantum Information and Quantum Physics, University of Science and Technology of China, Shanghai 201315, China;

3. Hefei National Laboratory, University of Science and Technology of China, Hefei 230088, China.

4. Department of Physics, The Chinese University of Hong Kong, Shatin, New Territories, Hong Kong SAR, China.

* Corresponding author. E-mail: yuminghe@mail.ustc.edu.cn; yongheng@ustc.edu.cn

CONTENTS

I. Details about the DBR-CBG structure 3

II. Sample growth and fabrication details 5

III. Optimization of passivation systems and techniques 7

IV. Improvements in non-resonant PL properties of QDs through the passivation 9

V. Reduction in the noise level of QD through the passivation 12

VI. Improvements in RF properties of QDs through the passivation 14

VI-I Dot-to-dot RF linewidth comparison 14

VI-II Statistical RF comparison on linewidth and wavelength 16

VII. Simulation on the band structure and surface electric field 18

VIII. Passivation influence on the wavelength of QDs' luminescence 19

IX. Summary of different surface passivation techniques employed on samples and corresponding XPS and Raman results 21

IX-I Summary of passivation process, XPS, and Raman results 21

IX-II Principles and results of Raman spectroscopy 22

X. Discussion of passivation effects in luminescence based on resonant and non-resonant excitation 24

XI. The original QD properties of the study 26

References 28

I. Details about the DBR-CBG structure

The DBR-CBG structure consists of 6 lateral circular gratings periods with 12 width parameters and 30 vertical pairs of λ/4-thick AlAs/GaAs DBR. The choice of 6 gratings is the balance between improvement in collection efficiency and decrement in fabrication uncertainties. The 12 width parameters of CBG are optimized by the bound optimization by quadratic approximation (BOBYQA) gradient algorithm to maximize the collection efficiency. The final parameters of the designed DBR-CBG structure are as follows.

Table S1: Parameters of the designed DBR-CBG structure.

Name	Designed value
Center ($2t_1$)	401.6 nm
Gap1 (d_1)	85 nm
Gap2 (d_2)	208 nm
Gap3 (d_3)	166 nm
Gap4 (d_4)	151 nm
Gap5 (d_5)	199 nm
Gap6 (d_6)	248 nm
Trench2 (t_2)	193 nm
Trench3 (t_3)	158 nm
Trench4 (t_4)	208 nm
Trench5 (t_5)	323 nm
Trench6 (t_6)	198 nm

As can be seen from Table S1, the diameter of the innermost circle is 401.6 nm. QDs are supposed to locate in the center based on 2 factors: The 1st one is from the CCD (charge-couple device) camera's image. The laser is focused on the center of the innermost circle according to our CCD camera's image in the experiment.

The 2nd one is in collection efficiency. If the QD is not at the center, according to our simulation results in Table S2, collection efficiency drops significantly when deviation is over 50 nm, making it difficult to detect RF signals. Therefore, considering all these factors, although the QDs may not be located in the absolute center, their lateral deviation will not be significant, making them less susceptible to the sidewall.

Table S2: Simulated collection efficiency of the DBR-CBG versus QD's lateral deviation.

Deviation distance	Simulated efficiency
0 nm	16.28%
50 nm	14.13%
100 nm	9.94%
150 nm	4.37%

According to our simulation, the quality factor (Q) equals 53 at the characteristic frequency of 336.65 THz (890.52 nm in vacuum). The mode volume equals $1.76*(\lambda_0/n)^3$, where λ_0 is the vacuum resonance wavelength and n is the GaAs bulk refractive index. The F_p factor is 2.29 (simulated) and 2.18 (measured), as illustrated in Figure S1. Considering the Purcell effect and radiation lifetime, Fourier transform limited linewidth with and without cavity is 870.98 MHz and 398.88 MHz, respectively.

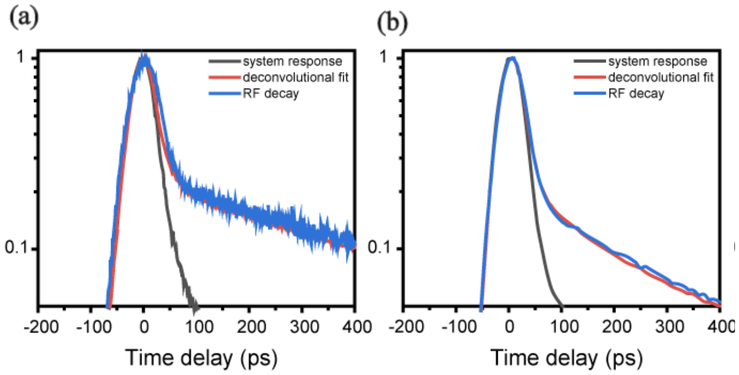

Figure S1. Radiative lifetime of the QD (a) outside and (b) inside the DBR-CBG structure. T_1 equals 399.00±11.50 ps and 182.73±3.90 ps for (a) and (b) respectively.

At last, we want to emphasize again that our motivation in the experiment is to use the DBR-CBG structure to enhance the collection efficiency of RF signals to investigate passivation improvements rather than design a high-performance nanocavity. The parameters of the structure have been carefully optimized, and based on the sample at hand, the collection efficiency structure is high enough for RF characterization in our experiment.

II. Sample growth and fabrication details

The fabrication process for the DBR-CBG structure, shown in Fig. S2 (a), consists of 3 main steps: sample growth, wet etching, and pattern definition (lithography and etch). The sample growth is performed as follows: First, we grow the epitaxial structure on a 3-inch GaAs (001) substrate, beginning with 30 pairs of 1 nm AlAs /3 nm GaAs super-lattice to obtain a smoother surface (not shown in the structure profile). Next, we grow 30 pairs of λ/4-thick AlAs/GaAs DBR to enhance vertical reflection. At last, we grow the source region composed of a λ-thick GaAs film with a layer of self-assembled InAs/GaAs QDs inserted in the middle.

After sample growth, we etch the surface GaAs utilizing a solution with a mixture of H_2O:H_2SO_4:H_2O_2 (volume ratio=50:1:1, etch rate ~1.1 nm s^{-1}). Previous works [1,2] have illustrated that the surface strongly degrades QD's optical properties when the dot-to-surface distance (*d*) is less than 40 nm. So, we etch to let *d* be 40 nm in this experiment. After etching, we perform X-ray diffraction (XRD) measurements to accurately determine *d*. Results of XRD analysis and corresponding fitting are illustrated in Fig. S2 (b), and *d* equals 38.20 nm according to the fitting results.

Subsequently, we define CBG patterns with electron-beam lithography (EBL) and inductively coupled plasma (ICP) etching processes. With the assistance of SEM measurement, we can obtain the exact value of lateral structure parameters and realize within ±10 nm deviation compared to designed parameters. Our sample consists of periodically arranged arrays of patterns and each pattern contains 2×7 CBG structures, as shown in Fig. S2 (c). With the location mark and the precise optical measurement setup, we can perform dot-to-dot comparisons. The surface morphology is illustrated by atomic force microscope (AFM) scanning in Fig. S2 (d), giving the 3D structure clearly.

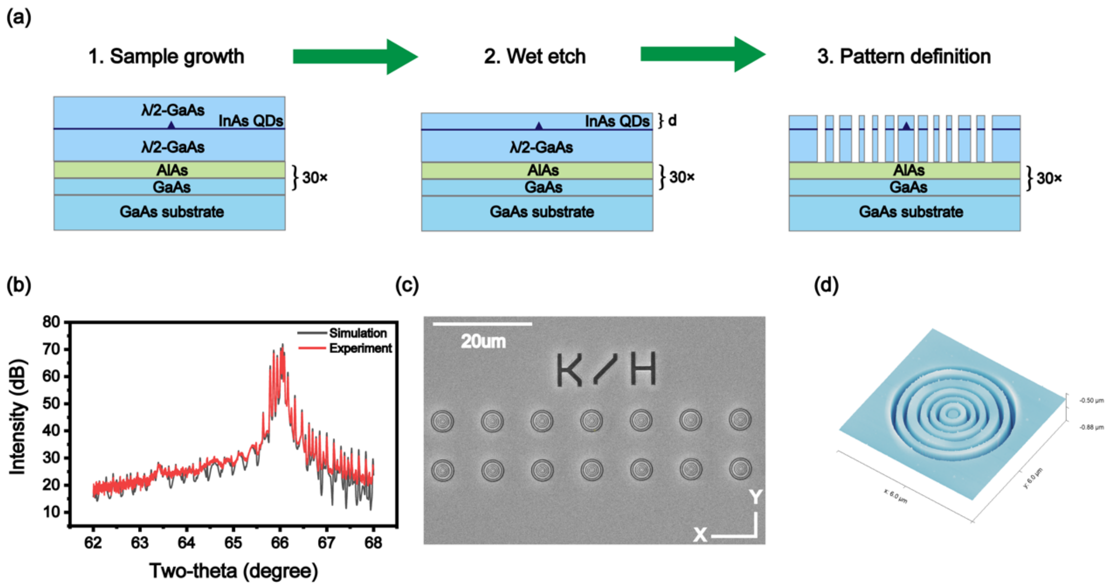

Figure S2. Sample growth and fabrication details of the DBR-CBG structure in the experiment. (a) Flowchart of the detailed sample fabrication process. (b) Results of XRD measurement and fitting of the data. (c) A top view of an SEM image of the structure in the X-Y plane. (d) An AFM image of the sample after fabrication, illustrating the 3D morphology of the DBR-CBG top structure (Image size: 6×6 μm, Oxford Instruments).

III. Optimization of passivation systems and techniques

Different passivation techniques are widely employed in not only modern semiconductor technologies but also in QD quantum light sources[1–4] as well. Just like ref [1–4] presented, there's no RF data both before and after passivation. Actually, we have tried these different techniques in the earlier period of our work, but we also can't observe any RF signals after passivation. The reasons can be either from the invalid passivation method itself or fast re-degradation (oxidation, etc.) processes directly after passivation. This is also the reason that we want to develop an optimal method to fulfill these requirements at the same time.

An effective passivation should eliminate the surface dangling bonds and protect the passivated surface from re-degradation simultaneously. In this way, we developed this optimal method to achieve these requirements at the same time.

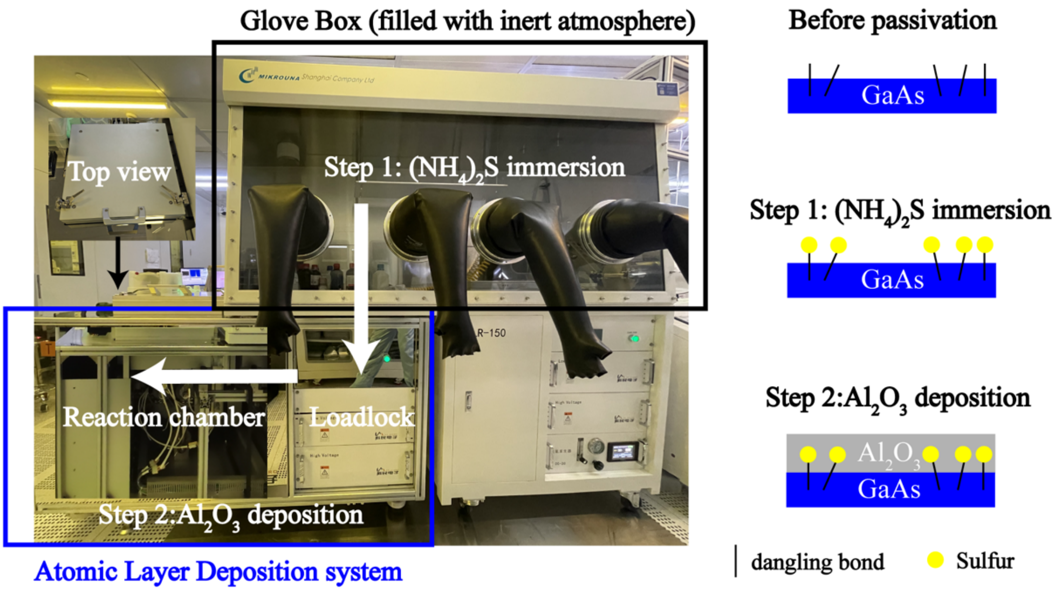

Figure S3. Schematic of our customized passivation system and passivation process. The whole process is conducted in an inert atmosphere or vacuum to preserve the sulfurization effects. The white arrows indicate the sample transfer direction.

Our passivation process is conducted in our customized system, as illustrated in Figure S3, consisting of a glove box connected to an Atomic Layer Deposition (ALD) system. The optimized passivation process is conducted as follows: First immersion in post-purified $(NH_4)_2S$ solution for 10 minutes in the glove box (H_2O and O_2

concentration<1 ppm), then safely transfer to the load-lock chamber of the ALD to deposit Al_2O_3.

Because the transfer is performed in an inert atmosphere, our method eliminates surface dangling bonds and protects the passivated surface from degradation simultaneously. In addition, as can be seen from Figure S4, stable and uniform passivation layers can only be obtained with the purification of $(NH_4)_2S$ solution and an inert atmosphere simultaneously.

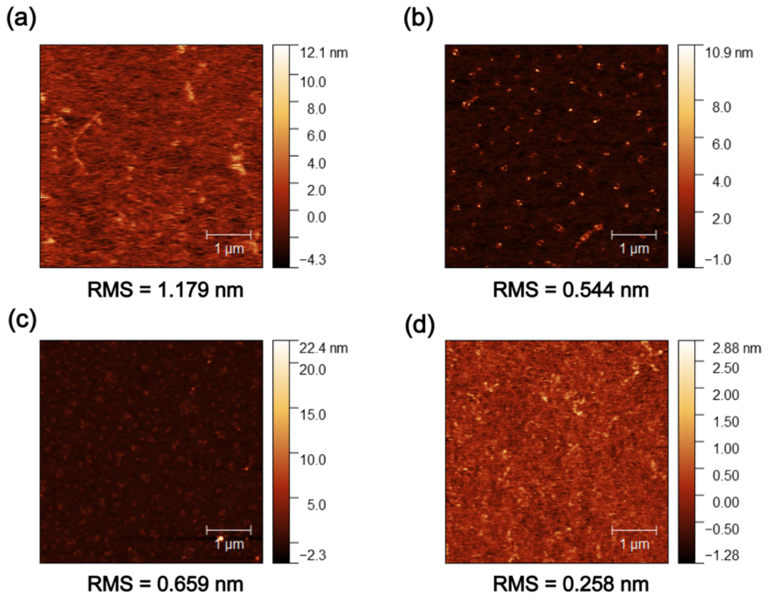

Figure S4. AFM images of GaAs surface (001) treated under different passivation conditions. Each sample is passivated by 20% $(NH_4)_2S$ immersion for 10 minutes followed by 10nm Al_2O_3 ALD deposition. The conditions that varied include whether the experiment is performed under an inert gas atmosphere and whether the $(NH_4)_2S$ solution is purified. (a) Without an inert atmosphere and purification. (b) With an inert atmosphere but without purification. (c) Without an inert atmosphere but with purification. (d) With an inert atmosphere and purification.

IV. Improvements in non-resonant PL properties of QDs through the passivation

In previous works, researchers randomly select tens of QDs before and after passivation and use the average linewidth to represent the quality of the PL of the sample. Decreased linewidth indicates improved optical properties through passivation. In this study, we randomly select 25 dots from the same sample before and after passivation respectively, and record their spectra using the same setup. The corresponding unitary PL spectra before and after passivation are illustrated in Figure S5 and Figure S6, respectively. The average linewidth decreases from 21.32±5.48 GHz to 16.49±2.03 GHz after passivation under non-resonant excitation.

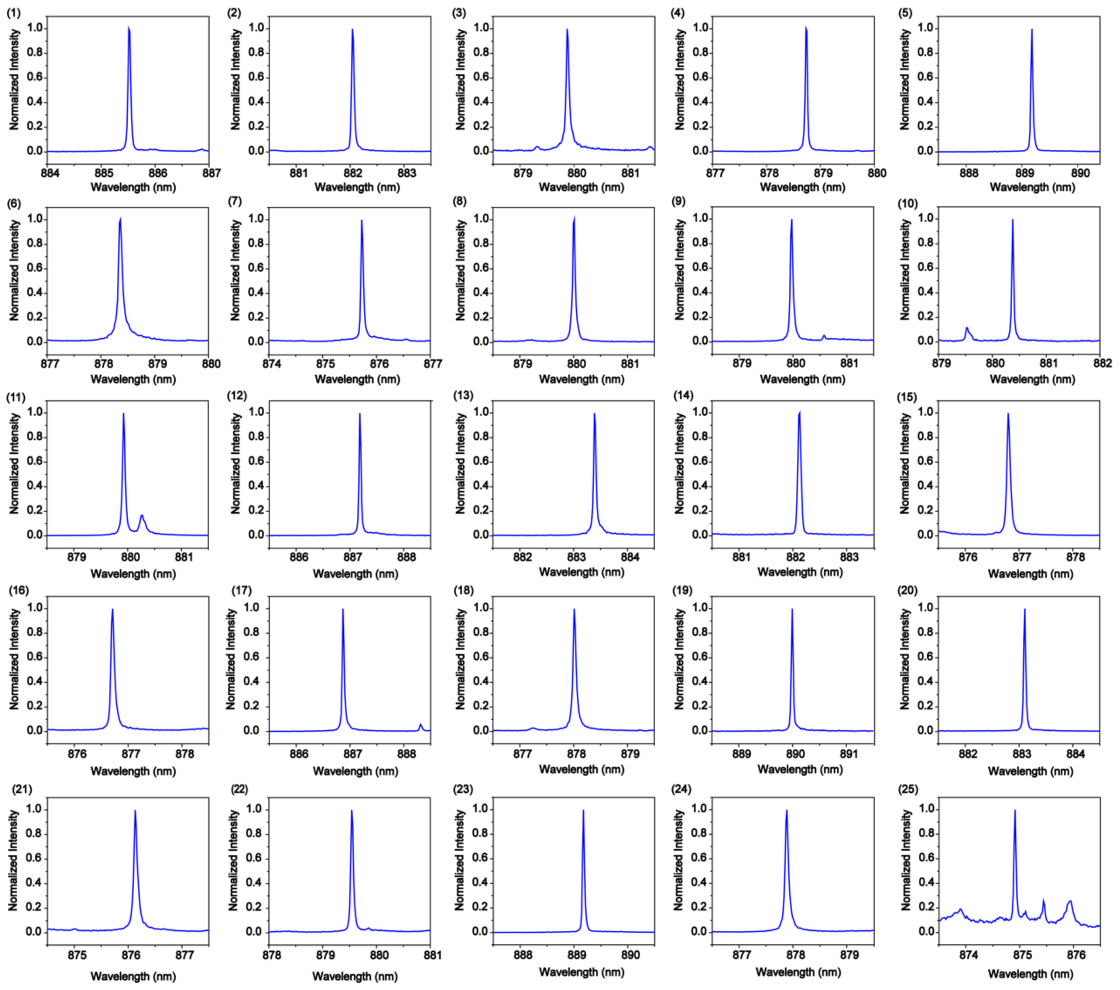

Figure S5. Non-resonant PL spectra of 25 randomly selected QDs before passivation.

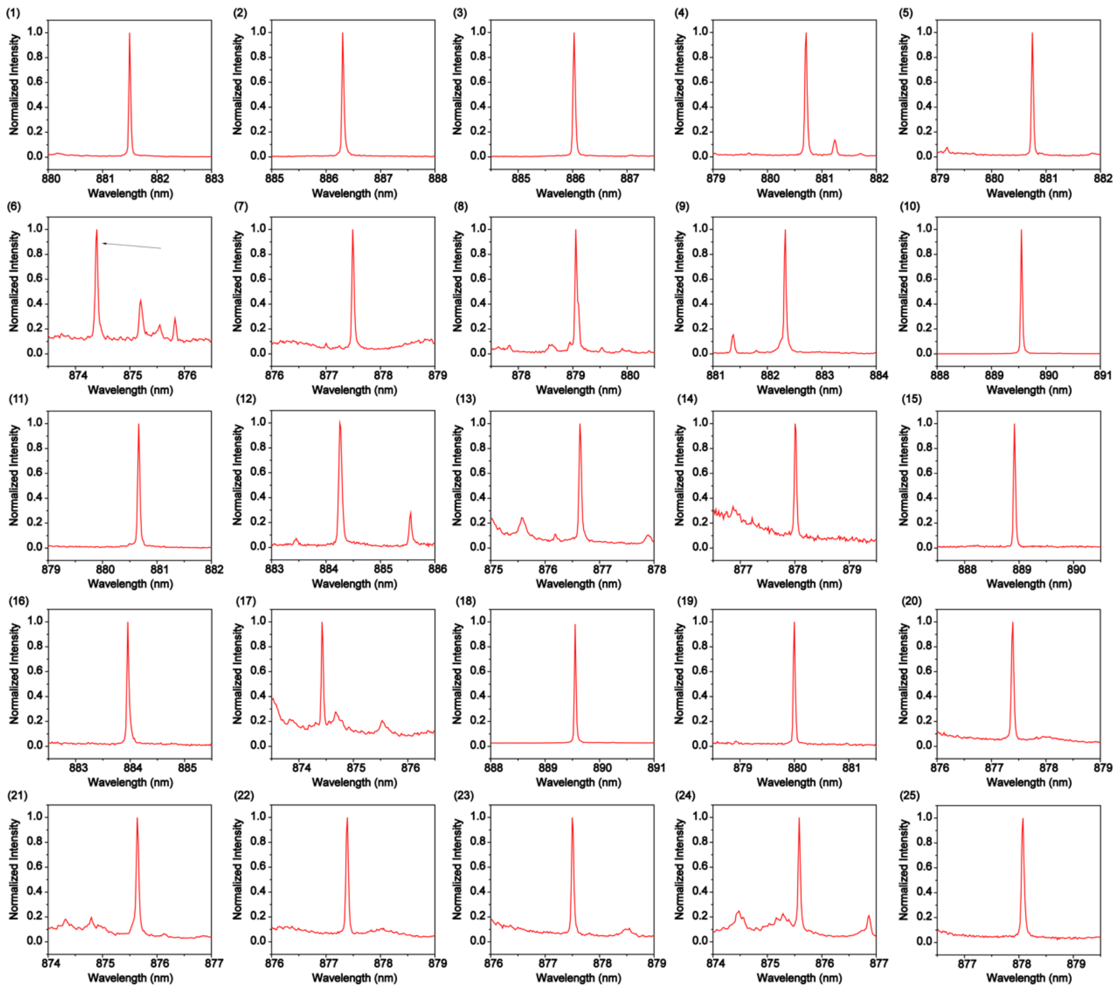

Figure S6. Non-resonant PL spectra of 25 randomly selected QDs after passivation.

V. Reduction in the noise level of QD through the passivation

Kuhlmann et al. used RF signals as a minimally invasive probe to investigate QD's intrinsic environment[5–8]. The intrinsic noise within the QD includes charge noise originating from occupation fluctuations of the defect states and spin noise arising from the fluctuations in nuclear spins of the host material. Charge noise introduces intensity blinking and linewidth broadening of the QD RF[9].

In this study, we attribute surface states to the main noise sources that degrade the optical properties of QDs. Hence the environment in the vicinity of QD should be stabilized after passivation. Previous works [5,8] use the Fourier transform of normalized RF signals as the noise spectra (eq.1). In this study, to comprehensively evaluate the noise level from QD, we choose the variance of the fluctuations as our criteria, which is equal to the integral of the total noise power (eq.2)[10]. We collect pulsed-RF signals from the QD and record the arrival time of each photon using an avalanche photodiode (APD), measure the noise level of QD and perform the dot-to-dot comparison.

$$N_{RF}(f) = \frac{\left|FFT\left[S(t)/\langle S(t)\rangle\right]\right|^2 (t_{bin})^2}{T} \qquad (eq.1)$$

$$\langle(\delta x)^2\rangle = \int_0^\infty df N_x(f) \qquad (eq.2)$$

Where t_{bin} is binning time selected, T is sampling time, $S(t)$ is the number of counts in each time bin, $\langle S(t)\rangle$ is the average number of counts per bin.

We choose QD2 (mentioned in the main article) as an example. After passivation, the variance of the fluctuations decreases from 0.2749 to 0.1587, indicating a 42.27% reduction in the total noise from QD2. The reduction in noise power of QD2 reveals a more stable environment in the vicinity of QD2, consistent with its improved RF properties.

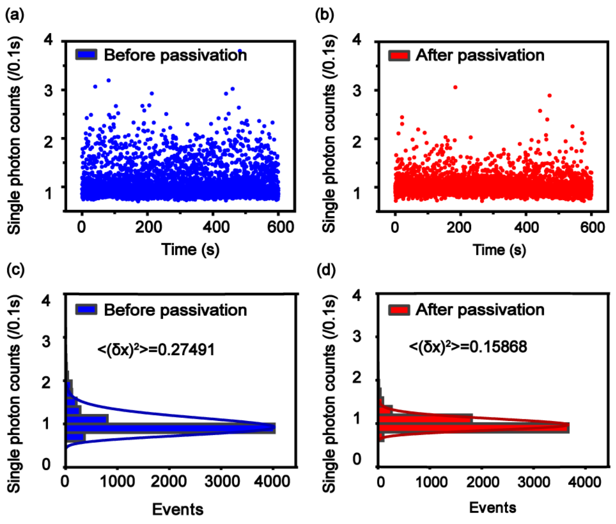

Figure S7. Reduction in the noise level of QD (QD2) through passivation. The spectrums are recorded over 10 minutes and transformed to the time domain (single photon flux) for better resolution. (a) and (b) correspond to single photon flux versus time before and after passivation, respectively. (c) and (d) correspond to the associated histogram before and after passivation, respectively.

VI. Improvements in RF properties of QDs through the passivation

In this part, we will provide detailed improvements in the RF properties of QDs through the passivation, including dot-to-dot comparison on RF linewidth (by Scanning Fabry–Pérot method) and statistical comparison on RF linewidth and wavelength (by spectrometer).

VI-I Dot-to-dot RF linewidth comparison

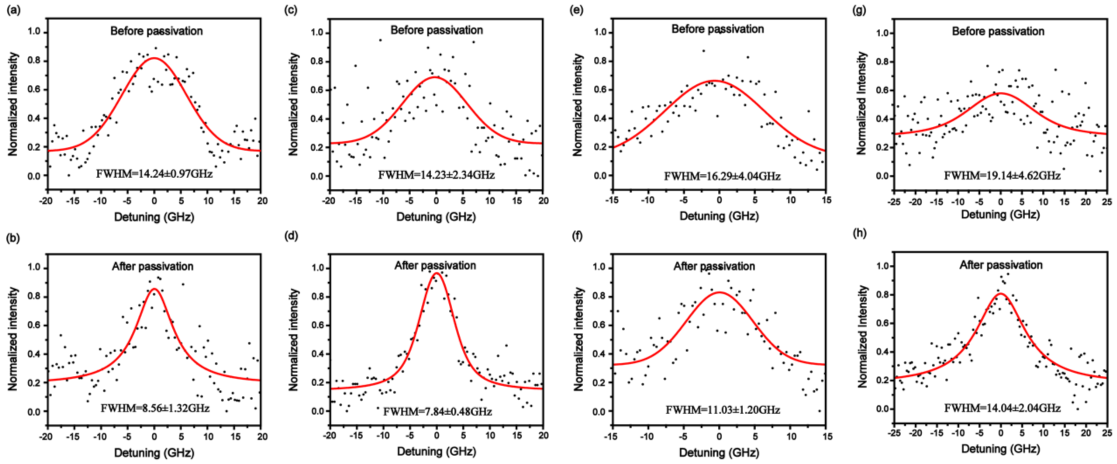

Figure S8. Comparison of the linewidth of pulsed-RF spectra from 8 QDs via the Scanning Fabry–Pérot (SFP) method before and after passivation (QD1-4). (a) and (b) correspond to QD1, (c) and (d) correspond to QD2, (e) and (f) correspond to QD3, (g) and (h) correspond to QD4.

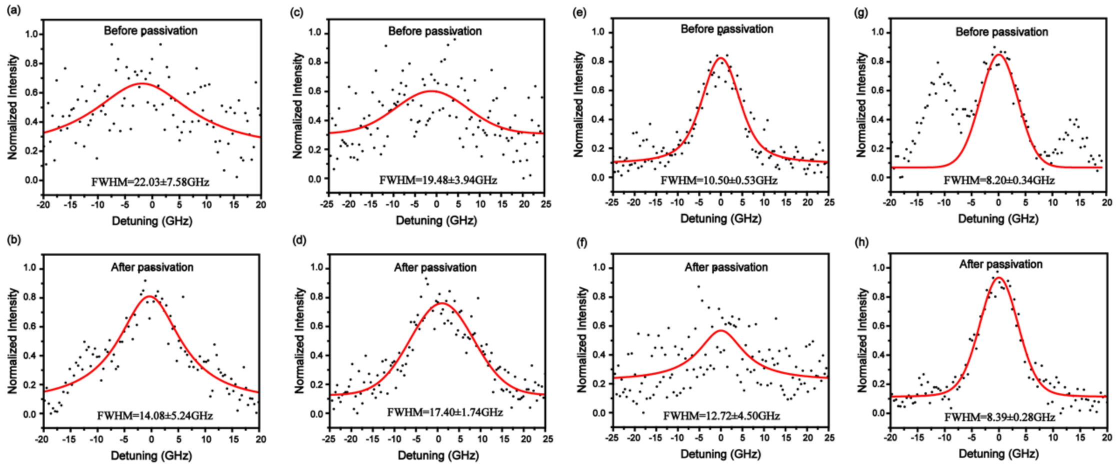

Figure S9. Comparison of the linewidth of pulsed-RF spectra from 8 QDs via the SFP method before and after passivation (QD5-8). (a) and (b) correspond to QD5, (c) and (d) correspond to QD6, (e) and (f) correspond to QD7, (g) and (h) correspond to QD8.

We can see from Fig. S8-S9 that obvious decrements in RF linewidth are observed after passivation. As for the deterioration effect on QD 7-8, we attribute it to newly generated defects arising from slight $(NH_4)_2S$ etching (rate ~0.363 nm h^{-1}) during passivation[11]. If the newly generated defects are located closely to the QD, the fluctuations of charge trapping and detrapping process will indeed have a strong impact on the QD and degrade its RF.

The impact can be estimated using eq.3, where *F* is the vertical electric field at the position of the QD created by a single positive charge at vertical distance *d* and lateral coordinate *r* from the QD. So, it's reasonable for QD 7-8 to show decrements of the properties after passivation. But statistically, the RF properties of most QDs will be improved after passivation. And that aligns with our experimental results.

$$F = \frac{-e}{4\pi\varepsilon_0\varepsilon_r}\frac{d}{(r^2 + d^2)^{\frac{3}{2}}} \qquad (eq.3)$$

We also notice that the RF linewidth signal contrast compared to the background is improved for QD 1-6 after passivation. We then define the signal-to-background ratio (SBR) as signal peak counts divided by background noise counts. As can be seen from Table S3, the SBR of most QDs (QD 1-6) increases after passivation. The drop in QD 7-8 aligns with their broadened linewidth, resulting from surface etching as mentioned above.

Table S3. Signal-to-background ratio of RF of 8 QDs in the main text.

QD Number	SBR value	
	**Before passivation**	**After passivation**
1	1.61	1.65
2	1.62	2.14
3	1.69	1.78
4	1.27	1.43
5	1.35	1.68
6	1.41	2.05
7	2.82	2.21
8	1.69	1.66

VI-II Statistical RF comparison on linewidth and wavelength

We also measure the RF spectra of 9 randomly selected QDs utilizing a spectrometer before and after passivation, respectively. The results are illustrated in Fig S10, where the average linewidth decreases from 43.23±22.53 GHz to 19.68±6.48 GHz, indicating a clear improvement after passivation. Meanwhile, the average center wavelengths lie in 878.57±2.62 nm and 880.76±3.36 nm before and after passivation, respectively, showing no apparent overall shifts in the wavelength.

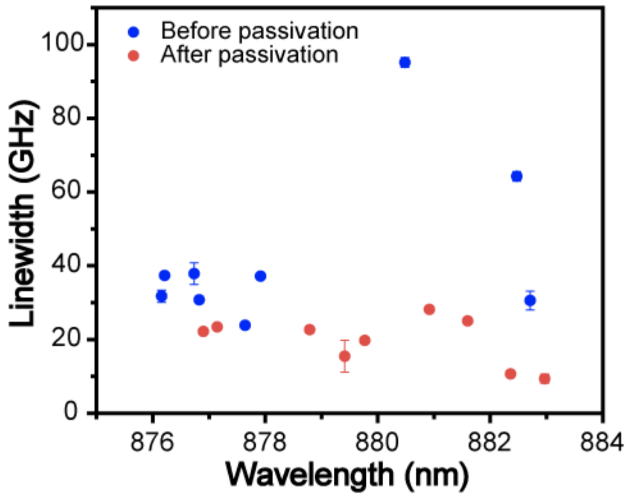

Figure S10. Comparison of the linewidth and wavelength of 9 randomly selected QDs before and after passivation under resonant excitation.

VI-III Dot-to-dot RF linewidth comparison on different capping thickness

To further confirm the validity of the simulations in Fig. 4, we have checked the influence of our passivation techniques for QDs with the dot-to-surface at ~127 nm. According to our simulation, the QD will not be influenced by the surface states at that depth, and the RF linewidth will be nominally the same before and after passivation. The experimental results are illustrated in Fig. S11. The RF linewidth equals 1.16±0.02 GHz before and 1.15±0.01 GHz after passivation, demonstrating no apparent change (slightly decrement within reasonable measurement error) and aligning with our simulations in Fig. 4.

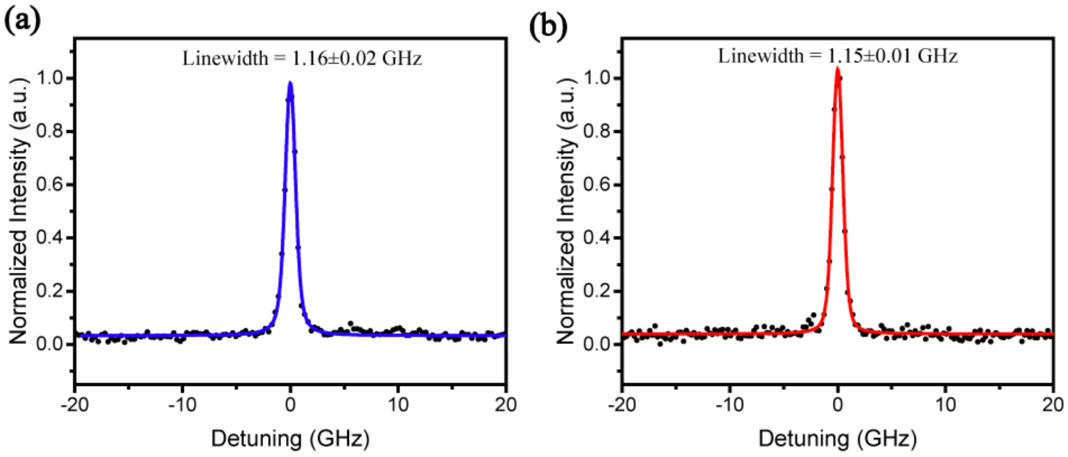

Figure S11. The linewidth of pulse-RF spectra from a single QD via the Scanning Fabry–Pérot (SFP) method (a) before and (b) after passivation.

VII. Simulation on the band structure and surface electric field

The 1D Schottky barrier model is often used to model the surface states[12]. This model treats the surface states as a Schottky-type contact with a given barrier height at the surface, and the barrier height reflects the density of surface states. In this work, we model the 1D device structure with the Schottky barrier in the top layer. We obtain the energy band structure and electric field distribution by solving the 1D Poisson equation numerically.

The simulated structure consists of a 0.1nm high P-doping GaAs (2×10^{20} cm^{-3}) at the surface. In the bulk GaAs, the P-doping is set at 3.2×10^{15} cm^{-3} according to our Electrochemical Capacitance-Voltage (ECV) result. The QD layer comprises a 1 nm $In_{0.2}Ga_{0.8}As$ layer. For the un-passivated case, we set the Schottky barrier at 1 *eV*[12], and 0.2 *eV* for the passivated case (verified by Raman results, see Supporting Information IX-I).

VIII. Passivation influence on the wavelength of QDs' luminescence

In this work, considering the passivation mechanism as reduced surface states and surface electric field, we want to investigate the passivation influence on the wavelength of QDs' luminescence (DC stark effect) in detail. We first investigate the average center wavelengths of the 25 randomly selected QDs, which lie in 881.04±4.68 nm and 880.62±4.59 nm before and after passivation, respectively (shown in Figure S12 (a)). The passivation process does not result in apparent overall shifts in the wavelength of PL of QD ensembles based on statistical analysis. The conclusion is similar for RF based on statistical analysis on 9 QDs in Supporting Information VI-II.

Subsequently, we turn to investigate the wavelengths of individual QDs before and after passivation. To achieve this, QD2 is chosen as an example.

$$\Delta E = a\Delta F \qquad (eq. 4)$$

$$\Delta F = \frac{\Delta V_g}{d} \qquad (eq. 5)$$

According to the reference [5], the shift on exciton energy E is determined by exploiting the DC-Stark effect linearly with electric field F and Stark shift coefficient a, which can be obtained by dividing the applied voltage ΔV_g by the thickness d.

We can easily obtain the Stark shift coefficient a based on our previous measurement on a P-I-N diode QD single-photon source. The source region is 223.3 nm thick, and the PL spectra shift for 0.2523 nm under 0.25 V voltage applied. Thus, the Stark shift coefficient a of our system is 0.0326 μeV•cm V^{-1}, close to the coefficient of neutral exciton in the previous article[5]. With a and shift magnitude of QD2 which illustrated in Figure S12 (b), we can easily obtain the reduced electric field intensity to be 10.1130 kV cm^{-1}, close to the simulated reduced electric field intensity (10.0686 kV cm^{-1}). This consistency further proves the validity of our proposed passivation mechanism.

Note: Here, the change magnitude of electric field intensity can be obtained with the formula below:

$$\Delta E = \frac{\int_{x_1}^{x_2} E(x)dx}{\int_{x_1}^{x_2} xdx} \qquad (eq. 6)$$

Where the $x_2 - x_1$ stands for the thickness of Bohr radius, $E(x)$ stands for the corresponding fitted electric field intensity.

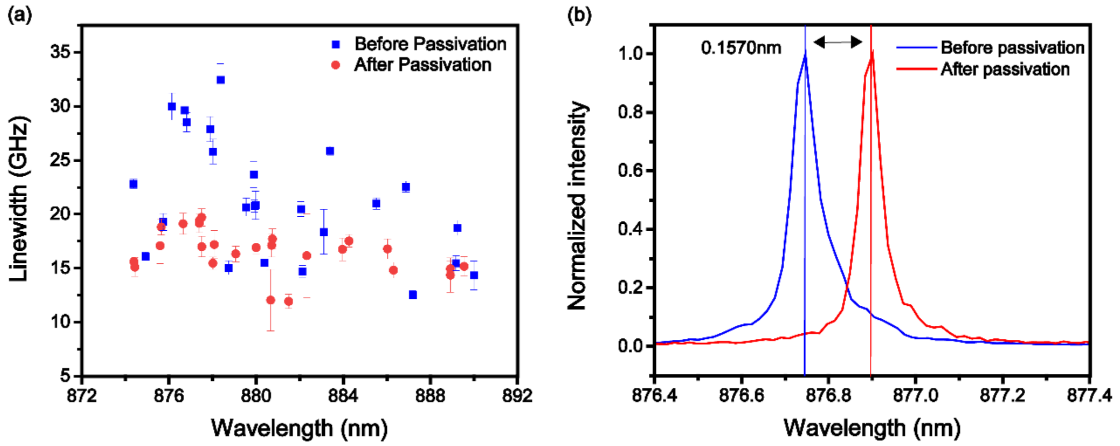

Figure S12. Passivation influence on the center wavelength of luminescence of QDs. (a) Comparison of wavelengths for 25 randomly selected QDs before and after passivation. (b) Pulsed-RF spectra of QD2 before and after passivation, demonstrating a slight redshift of 0.1570 nm on the wavelength after passivation.

IX. Summary of different surface passivation techniques employed on samples and corresponding XPS and Raman results

IX-I Summary of passivation process, XPS, and Raman results

Table S4. Summary of passivation process, XPS, and Raman results. Each surface treatment is conducted on a 10×10 mm GaAs piece cutting from the same 2-inch GaAs (001) substrate. The relative intensity changes of the peaks from XPS results reflect composition changes in surface oxides. Effective passivation should remove surface oxides completely. The Raman spectroscopy results reflect the ratio of thickness between the GaAs substrate and the surface depletion layer, expressed as I_{LOPC}/I_{LO} (LOPC: longitudinal-optic-phonon-plasmon coupled; LO: longitudinal optic, explained in the main article). The larger the value, the weaker the surface depletion layer signal, and thus the more effective the passivation is.

Surface Treatments	Process descriptions	XPS results	Raman results
Un-treated	/	Obvious Ga-O bond<br>Obvious As-O bond	I_{LOPC}/I_{LO} =0.798
ODT-passivation	Immersion in 0.05M L^{-1} ODT solution for 12 hours and drying naturally	Reduced Ga-O bond<br>Reduced As-O bond	I_{LOPC}/I_{LO} =1.362
ALD passivation	150℃ ALD 10 nm Al_2O_3	No Ga-O bond<br>No As-O bond	I_{LOPC}/I_{LO} =1.533
Dry-S +ALD passivation	100 ℃ 1 hour in H_2S atmosphere (UV-light), and transfer into ALD chamber to deposit 10 nm Al_2O_3 at 150℃	No Ga-O bond<br>No As-O bond	I_{LOPC}/I_{LO} =1.304
Wet-S +ALD passivation	Immersion in 20% $(NH_4)_2S$ solution for 10 mins, dry with N_2 gun, and transfer into ALD chamber to deposit 10 nm Al_2O_3 at 150℃	No Ga-O bond<br>No As-O bond	I_{LOPC}/I_{LO} =2.675

IX-II Principles and results of Raman spectroscopy

In this part, we provide more details about the I_{LOPC}/I_{LO} determined by Raman spectroscopy. Raman spectroscopy works by directing a monochromatic laser onto the sample, causing the molecules to scatter the light. The scattered light is then collected by a spectrometer, and the data is processed and analyzed to reveal information about the compositional and structural properties of the sample surface[13].

For the un-passivated sample, surface states introduce the surface electric field, resulting in surface band-bending. Surface band bending can lead to a reduction in the concentration of electrons/holes in the surface region, thereby forming a depletion layer. In this case, as long as the optical penetration depth is larger than the surface depletion layer thickness, Raman scattering can be observed from both the depletion layer and the bulk layer[14], as illustrated in Fig. S13. In the depletion layer, the scattering arises from the longitudinal optical (LO) phonons, where there are no free electrons for screening. In the bulk layer, the LO phonons couple with the free-electron plasmons and form the longitudinal-optic-phonon-plasmon coupled (LOPC) signals.

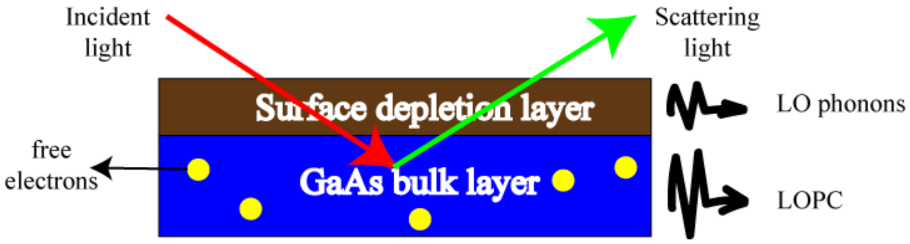

Figure S13. The schematic diagram illustrating the principle of I_{LOPC} and I_{LO} determined by Raman spectroscopy on the GaAs surface.

The intensity of I_{LO} and I_{LOPC} can be expressed as[15,16]:

$$I_{LO} = I_0(LO)\left(1 - e^{\frac{-2\delta}{D}}\right) \qquad (eq.7)$$

$$I_{LOPC} = I_0(LOPC)e^{\frac{-2\delta}{D}} \qquad (eq.8)$$

Where δ is the width of the surface depletion layer, D is the penetration depth of incident light, $I_0(LO)$ and $I_0(LOPC)$ are the intensity for undoped samples.

$$\frac{I_{LO}}{I_{LOPC}} = \frac{I_0(LO)}{I_0(LOPC)} \frac{1 - e^{\frac{-2\delta}{D}}}{e^{\frac{-2\delta}{D}}} \qquad (eq.9)$$

$$\delta = \left(\frac{\varepsilon_0 V_B}{2\pi e^2 n}\right)^{\frac{1}{2}} \qquad (eq.10)$$

Consequently, we can choose I_{LO}/I_{LOPC} to evaluate the degree of surface passivation. eq.9 can be obtained by dividing eq.7 by eq.8. eq.10 comes from ref [15,16], where n is the doping concentration, V_B is the surface induced barrier height, e is the electron charge, ε_0 is the static dielectric constant of GaAs. By measuring $\frac{I_{LO}}{I_{LOPC}}$ under different passivation techniques and divided by $\frac{I_{LO}}{I_{LOPC}}$ from un-treated samples, δ, and corresponding V_B can be obtained.

According to eq.10, $V_B = 1.0\,eV$ before passivation corresponds to $\delta = 8.96\,nm$. After passivation, δ reduces to $4.00\,nm$ due to $0.2\,eV$ barrier height. This change of δ will result in a 3.42-fold increment in $\frac{I_{LOPC}}{I_{LO}}$, consistent with the observed 3.35-fold increment in $\frac{I_{LOPC}}{I_{LO}}$.

X. Discussion of passivation effects in luminescence based on resonant and non-resonant excitation

In this part, we will discuss the difference of passivation effects on RF and non-RF. As is known to us, passivation aims to reduce the influence from surface states and provide a further capping layer to enhance device degradation resistance. Therefore, it's beneficial for both RF and non-RF. Non-resonant excitation is a fast, low-cost, and commonly used characterization technique. Without the need for a high-cost wavelength-tunable laser, it has been used in many labs, including ours, to do quick inspections. In general, only QDs with bright and sharp emission lines under non-resonant excitation will become candidates for further RF tests. For non-RF, surface passivation can eliminate surface states and enhance degradation resistance. But it cannot change the fundamental relaxation process. As can be seen from Fig. S14 (a), the inherent dephasing processes from higher energy states to the ground state generate phonons and restrict the properties of emitted photons, which are beyond the capability of passivation.

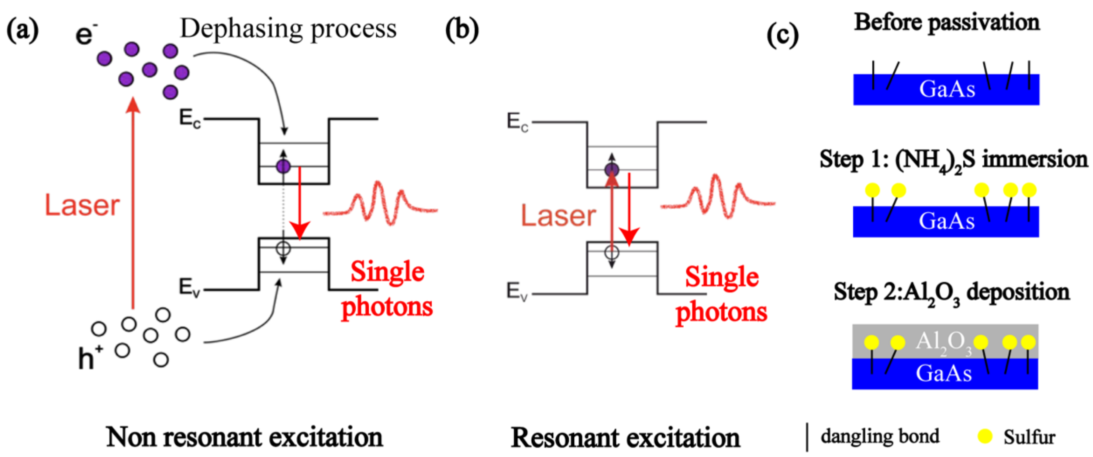

Figure S14. The principle of photons generation from (a) non-resonant and (b) resonant excitation. (c) The schematic of the passivation process.

Resonant excitation can produce high-quality single photons because it makes recombination happen directly between the two ground states without going through the inherent and noisy dephasing processes[17], as illustrated in Fig. S14 (b). For RF, surface passivation further shelters the two-level system from the influence of surface-

state level (within the bandgap) influence, making it cleaner and more stable. By resonantly exciting the QDs after passivation, improved RF properties is observed for near surface QDs. Consequently, good passivation (our in-situ two-step method) and excitation techniques (resonant excitation) are both required on the way to pursuing a superior thin film quantum light source.

XI. The original QD properties of the study

The original QD sample comes from a high-quality sample at hand for another experiment. The original dot-to-surface distance is ~127 nm, and the typical linewidth and lifetime of the QD lie in 1.16±0.02 GHz and 399.00±11.50 ps, as illustrated in Fig. S15 (a) and (b), and the linewidth is also above the Fourier-transform-limited.

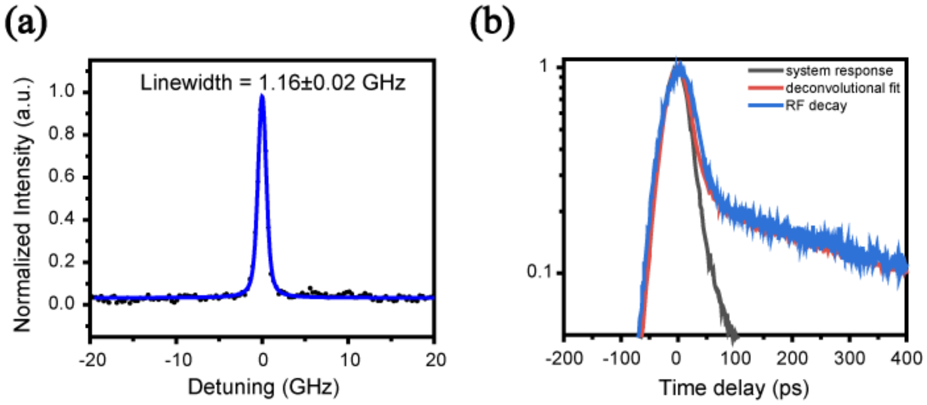

Figure S15. Original optical properties of the QD before etching. (a) High-resolution spectral characterization of emitted photons under π pulse excitation. The fitted linewidth equals 1.16±0.02 GHz. (b) The typical radiative lifetime of the QD equals 399.00±11.50 ps (deconvoluted value).

The excitons we studied in this experiment are positively charged. This is due to the background P-doping from the molecular beam epitaxy (MBE) setup, as can be seen from the Electrochemical Capacitance-Voltage (ECV) result in Fig. S16.

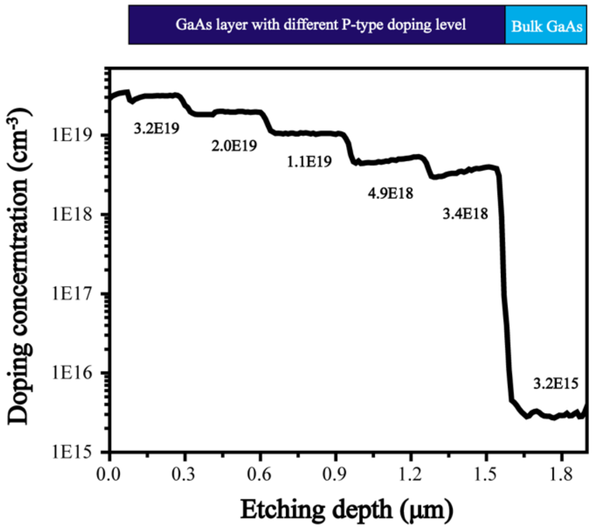

Figure S16. The Electrochemical Capacitance-Voltage (ECV) profile of our MBE grown sample, which indicates a 3.2E15 cm^{-3} P-type background doping level.

References

1 Manna S, Huang H, da Silva SFC, Schimpf C, Rota MB, Lehner B *et al.* Surface passivation and oxide encapsulation to improve optical properties of a single GaAs quantum dot close to the surface. *Appl Surf Sci* 2020; **532**: 147360.

2 Cao X, Yang J, Li P, Zhang Y, Rugeramigabo EP, Brechtken B *et al.* Single photon emission from ODT passivated near-surface GaAs quantum dots. *Appl Phys Lett* 2021; **118**.10.1063/5.0046042.

3 Chellu A, Koivusalo E, Raappana M, Ranta S, Polojärvi V, Tukiainen A *et al.* GaAs surface passivation for InAs/GaAs quantum dot based nanophotonic devices. *Nanotechnology* 2021; **32**.10.1088/1361-6528/abd0b4.

4 Liu J, Konthasinghe K, Davanco M, Lawall J, Anant V, Verma V *et al.* Single Self-Assembled InAs/GaAs Quantum Dots in Photonic Nanostructures: The Role of Nanofabrication. *Phys Rev Appl* 2018; **9**.10.1103/PhysRevApplied.9.064019.

5 Kuhlmann A V., Houel J, Ludwig A, Greuter L, Reuter D, Wieck AD *et al.* Charge noise and spin noise in a semiconductor quantum device. *Nat Phys* 2013; **9**: 570–575.

6 Houel J, Kuhlmann A V., Greuter L, Xue F, Poggio M, Warburton RJ *et al.* Probing single-charge fluctuations at a GaAs/AlAs interface using laser spectroscopy on a nearby InGaAs quantum dot. *Phys Rev Lett* 2012; **108**: 1–5.

7 Houel J, Prechtel JH, Kuhlmann A V., Brunner D, Kuklewicz CE, Gerardot BD *et al.* High resolution coherent population trapping on a single hole spin in a semiconductor quantum dot. *Phys Rev Lett* 2014; **112**: 1–5.

8 Zhai L, Löbl MC, Nguyen GN, Ritzmann J, Javadi A, Spinnler C *et al.* Low-noise GaAs quantum dots for quantum photonics. *Nat Commun* 2020; **11**: 1–8.

9 Lander GR. *Charge Dynamics of InAs Quantum Dots Under Resonant and Above-Band Excitation*. 2022.

10 Tomm N, Javadi A, Antoniadis NO, Najer D, Lobl MC, Korsch AR *et al.* A bright and fast source of coherent single photons. *Nat Nanotechnol* 2021; **16**: 399–403.

11 Uppu R, Midolo L, Zhou X, Carolan J, Lodahl P. Quantum-dot-based deterministic photon–emitter interfaces for scalable photonic quantum technology. *Nat Nanotechnol* 2021; **16**: 1308–1317.

12 Wang Y, Uppu R, Zhou X, Papon C, Scholz S, Wieck AD *et al.* Electroabsorption in gated GaAs nanophotonic waveguides. *Appl Phys Lett* 2021; **118**: 131106.

13 Pandey DK, Kagdada HL, Sanchora P, Singh DK. Overview of Raman Spectroscopy: Fundamental to Applications BT - Modern Techniques of Spectroscopy: Basics, Instrumentation, and Applications. In: Singh DK, Pradhan M, Materny A (eds). . Springer Singapore: Singapore, 2021, pp 145–184.

14 Chen X, Si X, Malhotra V. Measurement of Reduced Surface Barrier Height in Sulfur Passivated InP and GaAs Using Raman Spectroscopy. *J Electrochem Soc* 1993; **140**: 2085.

15 Farrow LA, Sandroff CJ, Tamargo MC. Raman scattering measurements of decreased barrier heights in GaAs following surface chemical passivation. *Appl Phys Lett* 1987; **51**: 1931–1933.

16 Wang J, Liu XH, Li ZS, Su RZ, Ling Z, Cai WZ *et al.* Raman scattering characterization of

the crystalline qualities of ZnSe films grown on S-passivated GaAs(100) substrates. *Appl Phys Lett* 1995; **67**: 2043.

17 He YM, He Y, Wei YJ, Wu D, Atatüre M, Schneider C *et al.* On-demand semiconductor single-photon source with near-unity indistinguishability. *Nat Nanotechnol* 2013; **8**: 213–217.
